# The synergy of *tuf* gene sequencing and maximum likelihood phylogenetic model: a suitable method for identifying the source of coagulase-negative *staphylococci* infections

**DOI:** 10.11604/pamj.2025.50.53.44554

**Published:** 2025-02-14

**Authors:** Innocent Afeke, Joseph Adu-Amankwaah, Lennox Mac Ankrah, Verner Ndudiri Orish, Ibrahim Jamfaru, Abdul-Wahab Mawuko Hamid, Kokou Hefoume Amegan-Aho, Hintermann Kobina Mbroh, Graceful Lord Mensah, Anthony Samuel Ablordey

**Affiliations:** 1 Department of Medical Laboratory Sciences, School of Allied Health Sciences, University of Health and Allied Sciences, Ho, Ghana,; 2 Department of Physiology, Xuzhou Medical University, Xuzhou, Jiangsu, China,; 3 Department of Microbiology and Immunology, School of Medicine, University of Health, and Allied Sciences, Ho, Ghana,; 4 Department of Pediatrics, School of Medicine, University of Health and Allied Sciences, Ho, Ghana,; 5 Department of Obstetrics and Gynaecology, Ho Teaching Hospital, Ho, Ghana,; 6 Department of Pediatrics, Ho Teaching Hospital, Ho, Ghana,; 7 Department of Bacteriology, Noguchi Memorial Institute for Medical Research, University of Ghana, Accra, Ghana

**Keywords:** *Tuf* gene sequencing, maximum likelihood phylogenetic model, *staphylococci(cal)* infections, neonates, clinical epidemiology, method

## Abstract

**Introduction:**

despite ongoing efforts, the health burden of neonatal infection remains unacceptably high due to major challenges, including the hurdle of determining the origin of infection. In this study, we explored the combination of tuf gene sequencing and the Maximum Likelihood Phylogenetic Model (MLPM) as a possible method for investigating the source(s) of transmission of two staphylococcal species, S. epidermidis and S. haemolyticus to neonates and young infants in the Ho Teaching Hospital (HTH) of Ghana, where we previously identified bloodstream infections as a major cause of neonatal morbidity and mortality.

**Methods:**

a total of 106 bacterial isolates were analyzed, comprising 67 S. epidermidis and 39 S. haemolyticus, cultured from blood samples of neonates and young infants, nasal mucosae of mothers, clinical staff, students, and a few objects in the hospital. Isolates were identified using Bruker Daltonik MALDI-TOF, and their nucleic acids were obtained. The tuf genes were sequenced using the Sanger method, and bioinformatics analyses were performed using the MEGA5 (10.1.8 version).

**Results:**

from our data, the combined use of bacterial tuf gene sequencing and MLPM revealed that mothers are the main source of S. haemolyticus-associated neonatal infections, whereas clinical staff is more likely to transmit S. epidermidis to neonates and young infants in the HTH. Whole genome sequencing scatter plots of a few of the isolates were used as a comparator method.

**Conclusion:**

overall, our findings suggest that using bacterial tuf gene sequencing in conjunction with bioinformatic analysis of this gene utilizing the MLPM may serve as a useful epidemiologic method in predicting the source of staphylococcal infections in neonates in the Neonatal Intensive Care Unit (NICU) and possibly other units in the hospital.

## Introduction

Healthcare-associated infections (HAIs) in neonates are a serious issue that contributes to increased morbidity and late mortality in the Neonatal Intensive Care Unit (NICU) of developing countries [1-3]. Studies show neonatal mortality rates resulting from HAIs in sub-Saharan Africa range from 17.0% to 29.0% [4]. In countries like Ghana, neonatal mortality is a huge problem. The latest national survey in 2011 estimated the country's mortality rate as 32 deaths per 1,000 live births [5], with some regions having a higher mortality rate than others. The Volta Region had the highest neonatal mortality rate of 47 deaths per 1,000 live births [6]. The Volta Region is one of Ghana's 16 administrative regions, with Ho as its capital. Previously, we have identified that bloodstream infections are a major cause of neonatal morbidity and mortality in Ghana's Ho Teaching Hospital (HTH) [7]. Undeniably, studies have shown that bloodstream infections (BSIs) are the most frequent HAIs in the NICU [1,2]. Due to several factors, up to 71% of neonates are vulnerable to bloodstream infections (BSI) while receiving intensive care [8].

In neonates and young infants, coagulase-negative staphylococci (CoNS) are the most frequent bloodstream bacteria responsible for late-onset infections [9,10]. However, the contribution of CoNS to neonatal sepsis may be influenced by socio-economic factors, as well as early-onset sepsis (EOS) and late-onset sepsis (LOS) [5]. CoNS exhibit multiple antibiotic resistance, and their ability to produce biofilm is thought to be the primary factor determining their pathogenicity. Clonal diversity among CoNS species varies and is much less studied than *Staphylococcus aureus*. There are a variety of approaches to analyzing these genomic data for epidemiologic and infection control purposes. Pain *et al*. have used comparative genome analysis to reveal key hospital adaptation and pathogenicity among *Staphylococcus haemolyticus* isolates cultured from a hospital setting [11]. At a high level, phylotyping may support epidemiological investigations to determine the source and routes of infections, trace cross-transmission of healthcare-associated pathogens, and identify virulent antibiotic-resistant lineages or subpopulations [12].

For CoNS, recent findings have shown that the *tuf* gene cluster in the short tandem repeat region on the bacterial chromosome shows significant diversity among them [6]. The *tuf* gene's small size and conserved location in bacterial chromosomes have contributed to its superiority in DNA sequencing compared with 16S rRNA for constructing a phylogenetic tree on species and genus level in staphylococci [13,14], especially in hospital settings. On this note, a study group has emphasized the value of partial *tuf* gene sequence to identify all staphylococcal species [6]. Comparative analysis of the *tuf* gene among staphylococci proved more discriminatory [6]. For CoNS, *tuf* gene sequencing provides a reference method with high accuracy for recognizing infections related to *S. epidermidis* and *S. haemolyticus* [15-18].

Increasing evidence reveals that the hands of healthcare workers [19,20], nasal mucosae of mothers [21], and the surface of objects [22] in the NICU may serve as the primary sources of neonatal infection; however, determining the distribution and transmission of pathogens, including CoNS to neonates and young infants with regard to healthcare workers, mothers, students and the surface of objects in the NICU remains a major challenge due to lack of suitable methods. Understanding the source(s) and transmission of the pathogens implicated in neonatal infections is pertinent to preventing and controlling HAIs in NICU. As such, we explored the combination of *tuf* gene sequencing and the Maximum Likelihood Phylogenetic Model (MLPM) as a possible method for investigating the source(s) of transmission of two staphylococcal species, *S. epidermidis* and *S. haemolyticus* to neonates and young infants in the HTH of Ghana.

## Methods

### Study definitions

***A neonate:*** a newborn baby in the first four (4) weeks after birth.

***Young infant:*** baby after four weeks to 12 months of age.

***Early-onset neonatal sepsis:*** a positive blood culture obtained within 72 hours of life.

***Late-onset neonatal sepsis:*** a positive blood culture obtained after 72 hours of life.

***Term born:*** baby born after 37 weeks of gestation.

***Preterm born:*** baby born at or before 37 weeks of gestation.

***Isolate labeled residence:*** isolate was obtained from a baby who was delivered (born) in the HTH and was admitted to the NICU of the Hospital. Also, isolate of the mother is labeled residence.

***Isolate labeled referral:*** isolate was obtained from a baby who was not born in the HTH but was referred from other districts or private hospitals. The mother's isolate is also labeled referral.

***The archived baby isolates:*** these were *S. epidermidis* and *S. haemolyticus* isolates cultured from babies' blood samples in the HTH in 2016, which were used in the current study in 2018.

***The study isolates:*** these were *S. epidermidis* and *S. haemolyticus* isolates cultured from babies' blood samples in 2018.

### Study site description

***Description of NICU in HTH:*** the NICU at the HTH, established in 2016 with a capacity of 22 beds, provides specialized care for preterm infants born before 28 weeks of gestation. Despite the unit's success in reducing the mortality rate from 22% to 19%, it faces significant challenges. The increasing number of preterm admissions, from 207 in 2019 to 338 in 2021 [23], underscores the growing demand for services. Staffing inadequacies are evident, with one nurse managing seven babies, and a sole doctor overseeing all 22 beds. Space constraints pose another hurdle, as the ward lacks sufficient room for proper cot spacing. Financial challenges compound the situation, as the cost of NICU services can be burdensome for mothers. Additionally, the NICU receives around 120 admissions every two months, including 35 referrals from other hospitals in the region and neighbouring cities in Togo.

**Study design:** the study was designed to explore the potential transmission of bacteria within the entire environment of neonates in the Ho Teaching Hospital's NICU. This open ward setting involved mothers using shared items. To encompass a comprehensive view, mothers of babies without positive blood cultures were also included. All on-duty clinical staff during the study period were eligible, with 59 out of 63 consenting to participate. Staff on leave but called for emergency duties were excluded. 96 mothers, including those with babies clinically diagnosed with sepsis or at risk, consented to participate, as previously detailed in our publication [24]. Bacterial isolates were collected from various participant categories, resulting in 527 isolates reported in our broader study. For the *tuf* gene investigation, we specifically focused on *S. epidermidis* and *S. haemolyticus*, common isolates associated with neonatal sepsis at HTH. Bacterial samples collected from the HTH Laboratory in 2016 were obtained through a convenience sample collection method. The laboratory manager provided *S. epidermidis* and *S. haemolyticus* cultured from neonates' blood samples. Revived from stored samples, these cultures were sent to Germany for further analysis. Only those identified as *S. epidermidis* and *S. haemolyticus* were utilized in the present study. We considered *S. epidermidis* implicated in early-onset neonatal sepsis as a potential contaminant, given its lack of clinical association with this condition. Additionally, blood cultures displaying mixed growth of skin flora were deemed possible contaminants.

**Bacterial culture from neonates and young infant blood samples:** bacterial cultures were performed in the HTH, University of Health and Allied Sciences (UHAS) Microbiology Laboratory, Ho, Ghana. Blood samples were collected by aseptic procedures and inoculated directly into BACTEC Peds Plus/F (Becton Dickinson Company, Maryland, USA) blood culture bottles for seven days. Blood samples were transported from the ward to the laboratory within 2 hours of being collected and placed in the BACTEC™ 9050 blood culture instrument. The culture bottles were incubated at 35°C with continuous agitation for a pre-determined time for maximum recovery of organisms. Positive culture bottles (Gram negative and Gram positive) were sub-cultured onto 5-10% sheep blood agar, harvested, and stored at -20°C in labeled sterile screw-cap vials until transported to Germany for further analyses. Susceptibility testing was done in Germany using a VITEK ™2 System.

**Figure 1:**
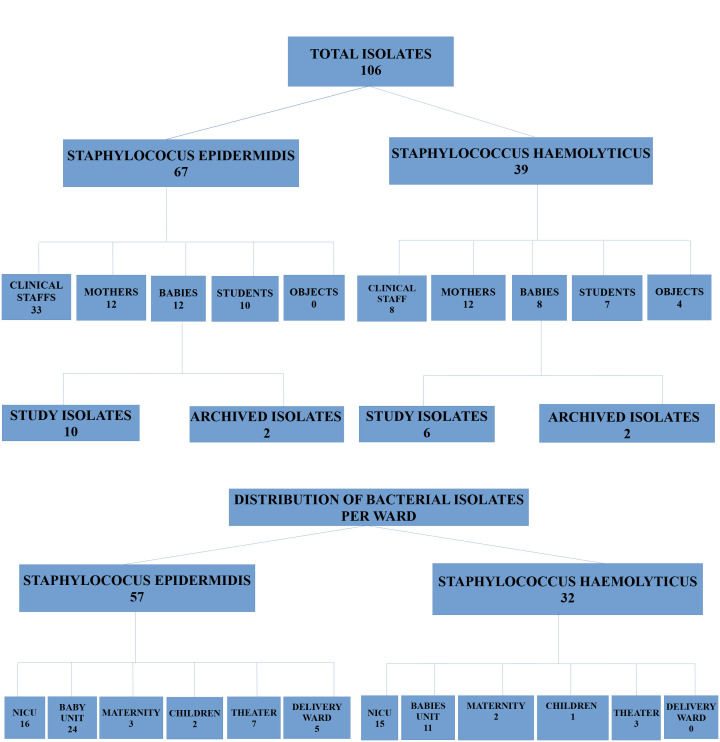
a flowchart showing the number of isolates from each category of participants and wards from which they were isolated

**Bacterial culture from swab samples from mothers, clinical staff, students, and objects:** nasal mucosae swab samples collected from the clinical staff, mothers, medical and nursing students (freshly admitted students who had no contact with the Hospital), and inanimate objects (babies' cots, incubators, weighing scales) were inoculated on 5% sheep blood agar plates and incubated aerobically at 37°C for 24 hours. The number of isolates from each category of participants and wards from which they were isolated is shown in the flow chart below (Figure 1). Bacterial cells were harvested and stored in Brain Heart Infusion (BHI) (Oxoid Ltd, England) with 15% glycerol at -20°C for further investigations.

**Identification of bacterial isolates:** all the bacterial isolates were identified in the Department of Infectious Diseases and Microbiology of the University of Lübeck, Germany, using the MALDI-TOF Biotyper G (Bruker Daltonics, Billerica, MA, USA). The bacterial isolates were revived from glycerol-preserved stocks by seeding them on a 5% - 10% sheep blood agar plate and incubated at 37°C for 24 hours or until visible growth was observed on the plate. The bacterial isolates were spotted from a single colony onto a MALDI-TOF MS 48-well target plate per the manufacturer's instructions and identified by the machine.

**DNA extraction:** DNA was extracted for 106 isolates using a DNeasy Blood and Tissue Kit (Qiagen, Germany). One sterile inoculation loop (~3mm^2^) of the plated overnight culture was resuspended in 100 µl of ultrapure water in a 1.5 ml microcentrifuge tube and mixed by pipetting. The bacterial DNA extraction protocol described in the kit for Gram-positive bacteria was followed without modification. The 100 µl bacterial suspension was centrifuged for 10 min at 7500 rpm, and the pellet resuspended in 180 µl enzyme lysis buffer (20 mM Tris-Cl pH 8.2, 2 Mm Na EDTA, 1.2% Triton®X-100, Lysozyme 20 mg/ml). The suspension was incubated for 30 min at 37°C. To this, 25 µl proteinase K and 200 µl buffer AL were added, mixed, and incubated at 56°C for 30 min. Then, 200 µl of molecular grade ethanol (96-100%) was added. The DNA extraction was then performed per the manufacturer's instructions.

**PCR amplification and *tuf* gene sequencing:** PCR amplification of the *tuf* gene was performed on a C1000 Touch™ Thermal Cycler (Bio-Rad) by applying a set of primers 5'-GCCAGTTGAGGACGTATTCT-3' and 5'-CCATTTCAGTACCTTCTGGTAA-3', which amplify a 412 bp fragment of the *tuf* gene [6]. PCR conditions were optimized as follows: a total reaction mixture of 50 µl contained 25 µl of DreamTaq Master Mix (10x buffer, 10mM dNTPs, and 5U/µl DreamTaq by Thermo Fisher Scientific, Bremen, Germany), 2.5 µl each of the primers, 18 µl of PCR water, and 2 µl of genomic DNA template. PCR was done in a DNA thermal cycler using the following program: 35 cycles with an initial denaturation at 95°C for 3 min, denaturation at 95°C for 30 sec, annealing at 56°C for 30 sec, extension at 72°C for 45 sec and final extension at 72°C for 10 min. PCR products of the *tuf* gene fragment with an estimated size of 412bp were separated by agarose gel electrophoresis. GeneRuler 100 bp Plus DNA ladder (Thermo Fisher Scientific) was used. PCR fragments were then assessed with the QUANTUM ST4 1100 image system (Vilber Lourmat Deutschland GmbH, Eberhardzell, Germany). The PCR products were aliquoted (46 µl) into 1ml Eppendorf tubes, sealed, and shipped to GENEWIZ-Brooks Life Sciences, Leipzig, Germany, for *tuf* gene sequencing. DNA sequencing was done by the Sanger method.

**Whole genome sequencing:** we conducted whole-genome sequencing on a randomly selected set of 14 isolates, which were subsequently compared using a scatter plot. This group includes *S. epidermidis* isolates from staff (HESMS03, HESMS05, HESMS10a, and HESMS13a), neonates (HESN021B, HESN016B, and HESN103B), and one from a mother (HESN034a). Additionally, five *S. haemolyticus* isolates were included, comprising staff isolates (HESMS016b, HESMS030a, and HESMS037) and neonate isolates (HESN068B and Baby076B). The whole-genome sequencing was conducted by the University of Kiel, Germany, utilizing next-generation sequencing technology.

**Bioinformatics analysis:** the obtained sequences of the *tuf* gene for each isolate were aligned separately by Bioedit, low-quality ends were trimmed, and all reads were made to have the same length. Data were transferred to MEGA 5 (Molecular Evolutionary Genetics Analysis) software and compared with all existing sequences of CoNS annotated in the GenBank database. A general phylogeny tree was generated, followed by the default set to draw pair sequences. Standard nucleotide BLAST was performed for all the *tuf* gene sequences at the website on Jan 27, 2020 [25]. The default was set for highly similar sequences (megablast) and searched standard databases. A total score of ≥600, query cover of ≥95%, and the highest identity of ≥97% were used to accept isolates at the species level. For the few isolates that whole genome sequencing was done, a scatter graph was determined using the raw sequenced data without assembly [26].

**Ethics approval and consent to participate:** the study was conducted in accordance with the Declaration of Helsinki. The Research Ethics Committee of the UHAS, Ghana, reviewed and approved this study with Protocol Identification Number UHAS-REC/A.2(1) 17-18. Written approval was also obtained from the HTH to use the facility for the study. Written informed consent was obtained from all the participants and mothers assented to their babies' involvement in the study.

## Results

***General description****:* we isolated 106 staphylococci in total, 67 were *S. epidermidis* and 39 *S. haemolyticus* (Figure 1).

### Maximum likelihood phylogenetic analysis of the tuf gene of S. epidermidis to determine clonal relatedness

**Figure 2:**
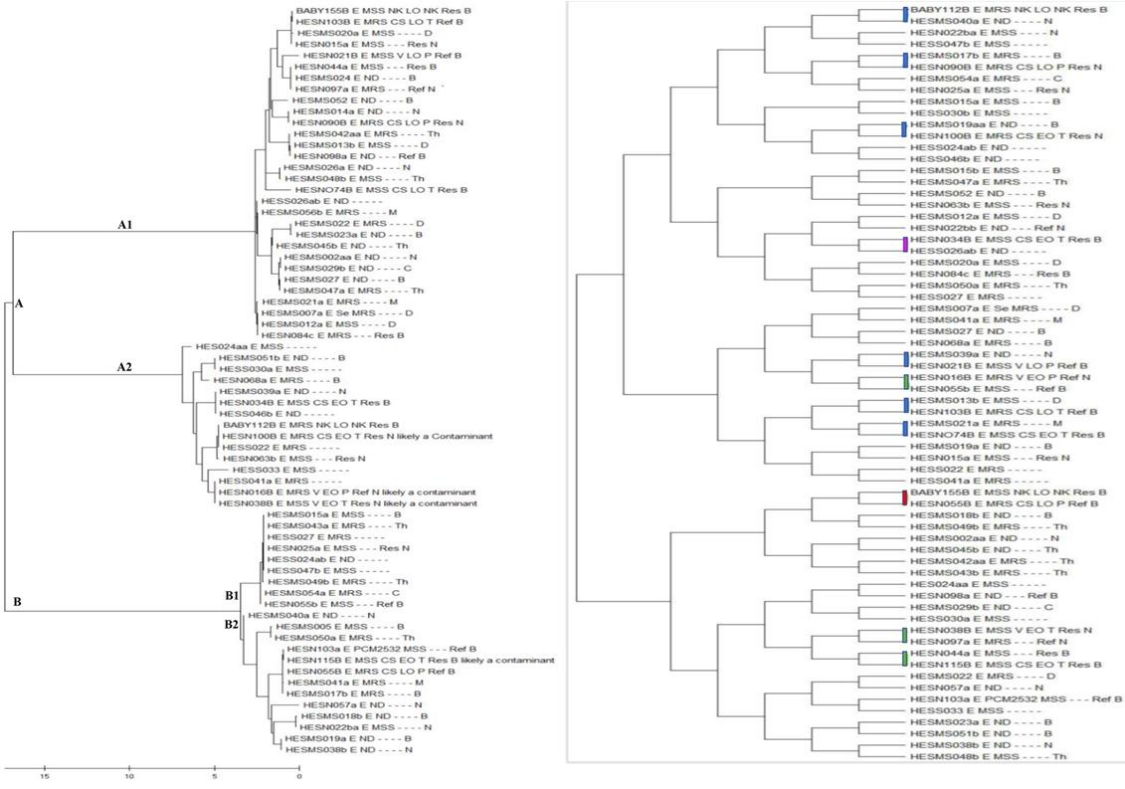
A) maximum likelihood phylogenetic analysis of the tuf gene of S. epidermidis to determine clonal relatedness (of note, no S. epidermidis isolate was cultured from the hospital environment); B) dendrogram showing pair-match of the 67 S. epidermidis tuf genes (blue: baby-clinical staff paired isolates; green: baby-mother paired isolates; pink: baby-student paired isolates; red: baby-baby paired isolates)

***General analysis of clusters:*** a dendrogram of the 67 *S. epidermidis* isolates cultivated from the HTH revealed two major clusters, labeled A and B (Figure 2A). Each major cluster has two sub-clusters indicated by A1, A2, and B1, B2.

**Analysis of clusters to predict possible hospital clones causing bloodstream infections:** analysis of percentages of the participants' isolates that fall into each sub-cluster showed that for each major cluster, a sub-cluster is more likely to have hospital clones that might be causing bloodstream infection among the neonates (sub-clusters A1 and B2). For other sub-classes, this is less likely (sub-clusters A2 and B1), as shown in Table 1.

**Table 1:** percentage distribution of S. epidermidis isolates in the major clusters and sub-clusters based on participant type

Comparing major cluster A with B	
Cluster A: 83% (10, n=12) BL; 62% (6, n=11) M; 66% (21, n=30) CS; 67% (6, n=9) S	Cluster B: 17% (2, n=12) BL; 38% (5, n=11) M; 38% (9, n=30) CS; 33% (3, n=9) S
**Comparing sub-cluster A1 with A2**	
**Cluster A1: 50% (5, n=10) BL; 67% (4, n=6) M; 90% (19, n=21) CS; 17% (1, n=6) S**	Cluster A2: 50% (5, n=10) BL; 33% (2, n=6) M; 10% (2, n=21) CS; 83% (5, n=6) S
**Comparing sub-cluster B1 with B2**	
Cluster B1: 0% (0, n=2) BL; 40% (2, n=5) M; 44% (2, n=5) CS; 100% (3, n=3) S	**Cluster B2: 100% (2, n=2) BL; 60% (3, n=5) M; 56% (5, n=9) CS; O% (0, n=3) S**

BL: baby; M: mother; CS: clinical staff; S: student. The bolded sub-clusters are likely hospital endemic clones.

**Pair analysis of isolates based on the similarities in their gene sequences:** the default for analysis was changed to identify and pair cluster isolate sequences that are highly similar. The following pair cluster was observed, as shown in Figure 2B.

**Percentage of pair-matched *tuf* genes of S. epidermidis isolates from babies' blood samples with other sources:** the study quantified the percentage of isolates paired with isolates cultivated from neonates' blood samples. It used pair-matched analysis for all 67 *tuf* gene sequences of the *S. epidermidis* isolates. The pair-match of neonates' blood isolates with isolates from nasal mucosae of clinical staff was estimated to be the highest, at about 55%. The pair-match of neonates' isolates with those of other neonates and students (control group) was the lowest, at about 9% (Table 2).

**Table 2:** percentage of pair-matched *tuf* genes of S. epidermidis isolates from babies' blood samples with other sources

Pair type	Number pair-matched (%) N=11
Isolates from the baby's blood pair-matched with isolates colonizing a mother	3 (27)
Isolates from a baby's blood pair-matched with another baby's blood isolates	1 (9)
Isolates from the baby's blood pair-matched with isolates colonizing a clinical staff member	6* (55)
Isolates from the baby's blood pair-matched with isolates colonizing a medical or nursing student	1 (9)

N: total number of pairs (percentages in parenthesis); *: four nurses (one male and three females) and two medical doctors (both males).

### Maximum likelihood phylogenetic tree obtained using the *tuf* genes of the 39 *S. haemolyticus*

**Figure 3:**
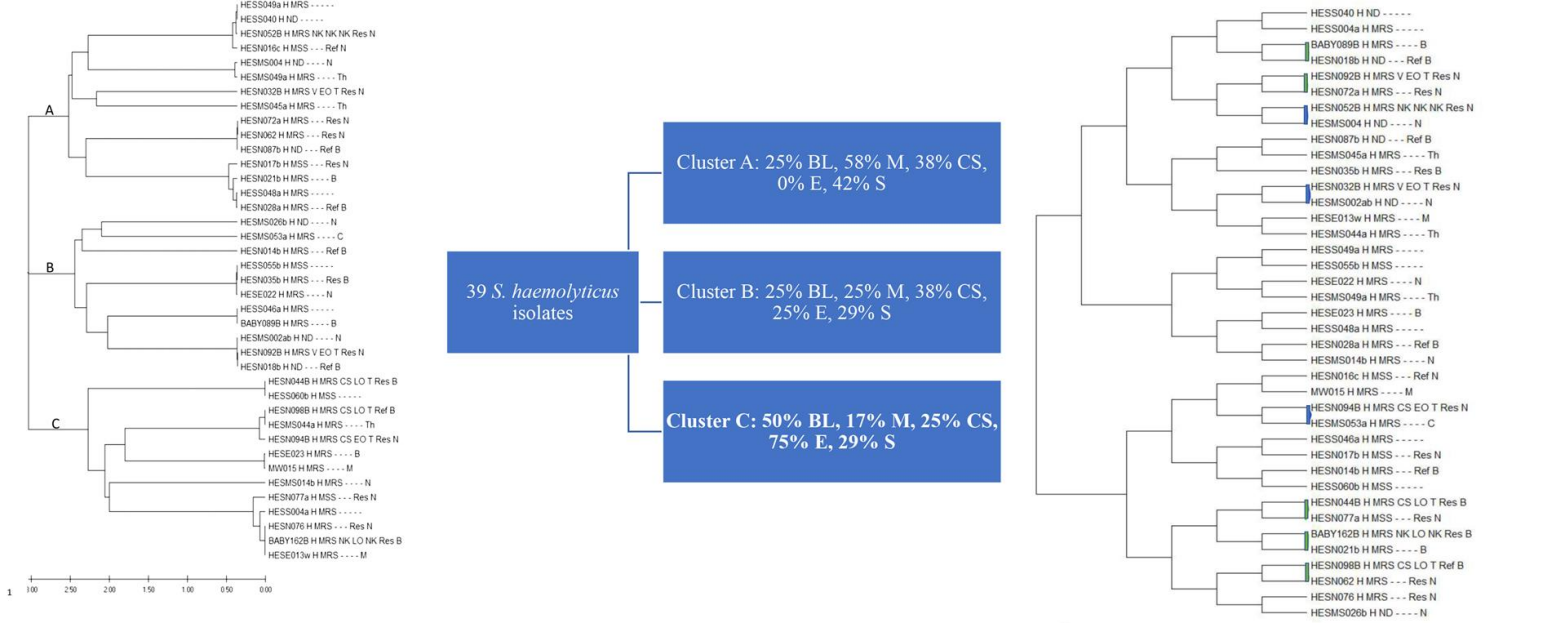
A) maximum likelihood phylogenetic tree obtained using the tuf genes of the 39 S. haemolyticus; B) percentage distribution of S. haemolyticus isolates into three major clusters based on participant type. BL: baby; M: mother; CS: clinical staff; S: student; E: environment; C) dendrogram showing pair-matched of the 39 S. haemolyticus tuf genes (blue: baby-clinical staff paired isolates; green: baby-mother paired isolate)

**General analysis of clusters:** for the 39 *S. haemolyticus* isolates analyzed, their *tuf* gene sequences dendrogram showed three major clusters, indicated as A, B, and C in Figure 3A, and each major cluster had about five sub-clusters.

**Percentages analysis among the major clusters:** the results showed that cluster C, with 50% of the neonates' isolates, 75% of isolates from an object in the hospital, and 25% of clinical staff's isolates, is more likely to be hospital-acquired transmission than the other two major clusters (Figure 3B).

**Pair analysis of isolates based on the similarities in their gene sequences:** pair analysis for the *S. haemolyticus* isolates revealed that five of the babies' isolates matched with the mothers' ones. Three of the babies' isolates matched with those of the clinical staff. Moreover, none of the babies' isolates matched with another baby's nor that of the students' isolates, as shown in Figure 3C.

**Percentage of pair-matched *tuf* genes of S. haemolyticus isolates from babies' blood samples with other sources:** the percentage of isolates paired with isolates cultured from blood samples of neonates was analyzed in the study. It used pair-matched analysis for all 39 *tuf* gene sequences of the *S. haemolyticus* isolates. The pair-match of neonates' blood isolates with isolates from nasal mucosae of mothers was estimated to be the highest, at about 63%. There was no pair-match of neonates' isolates with those of other neonates and students (control group) (Table 3).

**Table 3:** percentage pair-matched of *tuf* genes of S. haemolyticus isolates from babies' blood samples with other sources

Pair type	Number pair-matched (%) N=8
Isolates from the baby's blood pair-matched with isolate colonizing a mother	5 (63)
Isolates from a baby's blood pair-matched with another baby's blood isolates	0 (0)
Isolates from the baby's blood pair-matched with isolate colonizing a clinical staff member	3ɘ (38)
Isolates from the baby's blood pair-matched with isolates colonizing a medical or nursing student	0 (0)

N: total number of pairs (percentages in parenthesis); ɘ: two nurses (both female) and a medical doctor (male)

**Figure 4:**
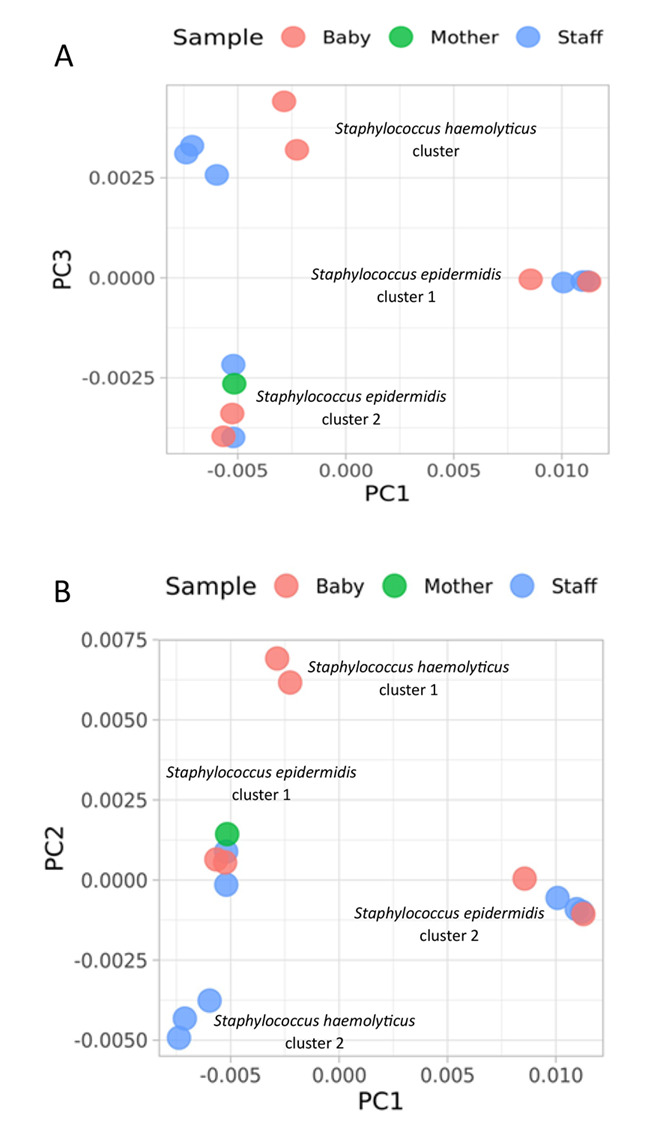
whole-genome scatter plots (A & B) of 14 isolates (9 *S. epidermidis* and 5 *S. haemolyticus*)

**Whole genome sequencing results:** heuristic cluster analysis using whole-genome sequences for the nine selected *S. epidermidis* and five *S. haemolyticus* showed an exact distance between the two staphylococcal species. For *S. epidermidis* isolates, two clusters were observed, with at least one isolate from clinical staff closely clustered with isolates cultivated from neonates' blood samples (Figure 4A and B). For *S. haemolyticus* isolates, although the neonates' isolates cluster together, they were relatively distanced from those of the clinical staff.

## Discussion

Despite the importance of CoNS as a cause of HAIs in neonates, little is known about their reservoirs, distribution, and mode of transmission within the NICU [27]. Due to a lack of effective methodologies, determining the distribution and transmission of CoNS to neonates and young infants with reference to healthcare professionals, mothers, students, and surfaces of objects in the NICU remains a significant hurdle. This study explored the combination of *tuf* gene sequencing and the MLPM as a possible method for investigating the source(s) of transmission of two staphylococcal species, *S. epidermidis* and *S. haemolyticus* to neonates and young infants in the NICU of HTH. We sequenced the *tuf* gene because, for CoNS, recent findings have shown that the *tuf* gene cluster, located in the short tandem repeat region on the bacterial chromosome, shows significant diversity among them [6]. Moreover, a comparative analysis of the *tuf* gene among staphylococci proved more discriminative. For CoNS, the *tuf* gene provides a reference method with high accuracy for recognizing hospital infections related to *S. epidermidis* and *S. haemolyticus* in critical care units [15,16]. Our current method, comprising of *tuf* gene sequencing in combination with MLPM, was able to highly discriminate the possible sources of transmission of *S. epidermidis* and *S. haemolyticus* isolates to neonates and young infants in the HTH.

For *S. epidermidis* isolates, *tuf* gene molecular analysis using MLPM demonstrated two major clusters that may represent the community's clones. The genetically heterogeneous isolates in the two major clusters may have originated from the native microbiota of individuals from the community. Each cluster had two sub-clusters, one of which was identified as a hospital-endemic clone. We considered this sub-cluster endemic clones because some isolates cultured from neonates' blood samples two years before the current study clustered with isolates from clinical staff and current neonates' isolates, indicating that they are endemic in the hospital environment. Similar work was done in Greece by Liakopoulos *et al*. [28], where they determine clonal relatedness of CoNS isolates recovered from hemodialysis patients' infections in a tertiary care center, and CoNS isolates colonizing clinical staff. The authors discovered one main clone of *S. epidermidis* that colonised 40% of the clinical staff and caused infection in the patients [28]. The method used in their study was Pulsed-field gel electrophoresis (PFGE). However, PFGE is no longer the preferred method for clonal relatedness. It has certain intrinsic disadvantages, such as being technically demanding and time-consuming, having poor reproducibility among different technicians, and being unable to differentiate between all unrelated isolates [29]. Moreover, PFGE results were reported to have correlated poorly with the actual relatedness of isolates [30-32], especially among more distantly related strains [33]. Therefore, there is a pressing need for new methods to replace the aforementioned method in clonal relatedness determination for bacterial species.

In our study, we found clonal similarity between nasal mucosae and bloodstream *S. epidermidis* isolates for two sub-clusters of five out of thirty and two out of thirteen isolates using bacterial *tuf* gene sequencing in combination with MLPM as a possible method. Our findings were in line with a study conducted by Soeorg *et al*. [34-36], where a multilocus variable number of tandem repeats analysis was used as a method to determine the genetic relatedness of *S. epidermis* isolates from the gastrointestinal tract (GIT) and blood samples of preterm neonates with late-onset sepsis. Their data showed clonal similarity between the GIT and bloodstream *S. epidermidis* isolate of three out of seven isolates; however, the method from our study identified and compared the clonal relatedness between isolates from neonates and their environment (i.e., staff, mothers, and objects in the NICU), whiles the method from Soeorg *et al*. [37] study determined clonal similarity between isolates from GIT and bloodstreams of the same neonates. Not all molecular methods used to determine possible sources of CoNS infections have reported similarity of bacterial clones; for instance, the method of DNA restriction fragment polymorphism used by Kacica *et al*. [35] to determine the relatedness of CoNS causing bacteremia in low birthweight infants, showed that those bacterial species with similar restriction fragment length patterns could not be linked as nosocomially transmitted among infants with bacteremia.

For *S. haemolyticus*, our current method obtained three major clusters for the 39 *S. haemolyticus* isolates' *tuf* genes analyzed. However, one of the major clusters, with 50% of the neonates' isolates and 75% of isolates from objects in the hospital, is likely to be the hospital-endemic clones. In NICUs, a prior study had described the use of southern blot hybridization of ClaI digests with mecA DNA probes and PFGE of SmaI digest of chromosomal DNA to demonstrate the association between disease and colonization among preterm neonates and single clones of multi-resistant *S. haemolyticus* strains that produce biofilms [36]. Multilocus sequence typing by PFGE for *S. haemolyticus* isolated from GIT and blood of preterm neonates with late-onset sepsis identified ten out of eleven isolates to be genotypically similar [34]. Their current study using the same method to investigate clonal relatedness of *S. haemolyticus* isolated from preterm neonates' gut, skin, and milk of their mothers revealed that most frequent multilocus variable number tandem repeat analysis types belonged to sequence type 3 or 42, comprised 71.1%-78.4% of isolates from preterm neonates/mothers and caused all seven late-onset sepsis episodes [37]. Whole genome sequencing and single nucleotide polymorphisms in the core genome of *S. haemolyticus* isolates from neonatal blood cultures at a Swedish NICU over four decades showed a clonal outbreak of this staphylococcal species at the NICU during the 1990s and that the isolates in the outbreak were more homogenous in their genotypic patterns [38]. The use of whole genome sequencing to determine clonal relatedness is the gold standard; however, it is expensive when many isolates need to be analyzed. An equally less expensive will be desirable for analyzing bacterial species relatedness by hospital epidemiologists.

The ability of our method to pair *tuf* gene sequences of two highly related genes among major and minor clusters will help identify the potential source(s) of infection within NICUs and other wards in the hospital, as reported by many studies [28,39]. Within - and inter-wards transmission of CoNS can be predicted to help formulate measures to reduce HAIs. From our data, the combined use of bacterial *tuf* gene sequencing and MLPM revealed that mothers are the main source of *S. haemolyticus*-associated neonatal infections, whereas clinical staff is more likely to transmit *S. epidermidis* to neonates and young infants in the HTH. There is a paucity of data describing mothers or clinical staff as possible sources of *S. epidermidis* and *S. haemolyticus* transmission in NICUs. However, bacterial ecological studies have reported that *S. epidermidis* is found ubiquitously on human skin [38] while *S. haemolyticus* is also part of the skin flora of humans, but its largest population is usually found at the axillae, perineum, and inguinal areas [40]. While the clinical staff is most likely to have hand contact with neonates, neonates' mothers stand the chance of their other body parts coming into contact with their babies. Findings from our current method support this theory and therefore call for more research in reproducing the results of predicting possible sources of transmission of CoNS and possibly other bacterial species in various wards in the hospital.

**Limitations:** while our method holds promise for identifying sources of transmission in CoNS infections, it is important to recognize that our study has certain limitations. Specifically, we did not directly compare all our isolates with established molecular typing methods like MLST or WGS. However, our examination of a limited number of isolates through whole-genome sequencing revealed that *S. haemolyticus* isolates from neonates' blood samples exhibited notable divergence from isolates colonizing clinical staff, as indicated by the whole-genome scatter plot. Unfortunately, our analysis does not include isolates from mothers, preventing us from drawing conclusions about their relatedness. Regarding *S. epidermidis*, some neonatal isolates appear to cluster with those from the staff, aligning with findings from the *tuf* gene method we employed. However, we exercise caution in interpreting these results due to the limited number of isolates subjected to whole-genome sequencing. Additionally, the small size of the sequenced reads has precluded genome assembly. Future research could benefit from such comparative analyses to better assess the effectiveness of our proposed method. Additionally, though we collected data on the specific wards where participants were located, the admission types of babies and their mothers, the delivery method for babies, and the methicillin susceptibility of the isolates, we have yet to investigate their potential associations with the phylogenic clusters of the isolates. Therefore, further studies are required to explore these aspects. Also, other limitations of our study, including the relatively small sample size, the absence of in-depth epidemiological data, and the lack of a robust comparator should be acknowledged. To bolster the validity of any conclusions regarding the sources of these bacteria, we underscore the importance of future research with larger sample sizes and a comprehensive approach, encompassing detailed epidemiological data and a more robust comparator.

## Conclusion

Our findings suggest that using bacterial *tuf* gene sequencing in conjunction with bioinformatic analysis of this gene utilizing the MLPM may serve as a useful hospital epidemiological method in predicting the source(s) of staphylococcal infections in neonates in the NICUs. More research is needed to reproduce the results of this method in other bacterial species and various wards in the hospital to inform measures to reduce HAIs, especially in geographic areas that are mostly affected.

### What is known about this topic

- Coagulase-negative staphylococci, particularly S. epidermidis and S. haemolyticus, are common causes of neonatal infections;
- Determining the origin of these infections in neonatal intensive care units (NICUs) is challenging, impacting infection control measures;
- Molecular methods like tuf gene sequencing have been utilized for bacterial identification and epidemiological studies.

### What this study adds

- The combination of tuf gene sequencing and the Maximum Likelihood Phylogenetic Model (MLPM) can effectively identify the source of neonatal infections in a hospital setting;
- The study identified mothers as the primary source of S. haemolyticus infections and clinical staff as the main source of S. epidermidis infections in neonates;
- This method provides a practical tool for epidemiological investigations and could aid in targeted infection control strategies in NICUs.

## References

[1] Sass L, Karlowicz MG (2018). Healthcare-Associated Infections in the Neonate: Principles and Practice of Pediatric Infectious Diseases.

[2] Ceparano M, Sciurti A, Isonne C, Baccolini V, Migliara G, Marzuillo C (2023). Incidence of Healthcare-Associated Infections in a Neonatal Intensive Care Unit before and during the COVID-19 Pandemic: A Four-Year Retrospective Cohort Study. J Clin Med.

[3] Frantzis I, Levasseur S, Huebner J, Mahida M, Larussa P, James W (2024). Infection prevention and control and related practices in African neonatal units: The Pan-African neonatal care assessment study (PANCAS). Int J Hyg Environ Health.

[4] Ranjeva SL, Warf BC, Schiff SJ (2018). Economic burden of neonatal sepsis in sub-Saharan Africa. BMJ Glob Health.

[5] Adjei G, Darteh EKM, Doku DT (2021). Neonatal mortality clustering in the central districts of Ghana. PLoS One.

[6] Khosravi AD, Roointan M, Abbasi Montazeri E, Aslani S, Hashemzadeh M, Taheri Soodejani M (2018). Application of *tuf* gene sequence analysis for the identification of species of coagulase-negative staphylococci in clinical samples and evaluation of their antimicrobial resistance pattern. Infect Drug Resist.

[7] Afeke I, Hirose M, Amegan-Aho KH, Haertel C, Becker M, Moustafa A (2021). Neonatal and Young Infant Sepsis in a Regional Hospital in Ghana. Open Journal of Pediatrics.

[8] Mpinda-Joseph P, Anand Paramadhas BD, Reyes G, Maruatona MB, Chise M, Monokwane-Thupiso BB (2019). Healthcare-associated infections including neonatal bloodstream infections in a leading tertiary hospital in Botswana. Hosp Pract 1995.

[9] Klingenberg C, Aarag E, Rønnestad A, Sollid JE, Abrahamsen TG, Kjeldsen G (2005). Coagulase-negative staphylococcal sepsis in neonates. Association between antibiotic resistance, biofilm formation and the host inflammatory response. Pediatr Infect Dis J.

[10] França A (2023). The Role of Coagulase-Negative Staphylococci Biofilms on Late-Onset Sepsis: Current Challenges and Emerging Diagnostics and Therapies. Antibiotics (Basel).

[11] Pain M, Hjerde E, Klingenberg C, Cavanagh JP (2019). Comparative genomic analysis of Staphylococcus haemolyticus reveals key to hospital adaptation and pathogenicity. Front Microbiol.

[12] Schürch A, Arredondo-Alonso S, Willems R, Goering RV (2018). Whole genome sequencing options for bacterial strain typing and epidemiologic analysis based on single nucleotide polymorphism versus gene-by-gene-based approaches. Clin Microbiol Infect.

[13] Hwang SM, Kim MS, Park KU, Song J, Kim EC (2011). Tuf gene sequence analysis has greater discriminatory power than 16S rRNA sequence analysis in identification of clinical isolates of coagulase-negative staphylococci. J Clin Microbiol.

[14] Shin JH, Kim SH, Jeong HS, Oh SH, Kim HR, Lee JN (2011). Identification of coagulase-negative staphylococci isolated from continuous ambulatory peritoneal dialysis fluid using 16S ribosomal RNA, tuf, and SodA gene sequencing. Perit Dial Int.

[15] Capurro A, Artursson K, Waller KP, Bengtsson B, Ericsson-Unnerstad H, Aspán A (2009). Comparison of a commercialized phenotyping system, antimicrobial susceptibility testing, and tuf gene sequence-based genotyping for species-level identification of coagulase-negative staphylococci isolated from cases of bovine mastitis. Vet Microbiol.

[16] Alexopoulou K, Foka A, Petinaki E, Jelastopulu E, Dimitracopoulos G, Spiliopoulou I (2006). Comparison of two commercial methods with PCR restriction fragment length polymorphism of the tuf gene in the identification of coagulase-negative staphylococci. Lett Appl Microbiol.

[17] Chiquet C, Musson C, Aptel F, Boisset S, Maurin M (2018). Genetic and Phenotypic Traits of Staphylococcus Epidermidis Strains Causing Postcataract Endophthalmitis Compared to Commensal Conjunctival Flora. Am J Ophthalmol.

[18] Bergeron M, Dauwalder O, Gouy M, Freydiere AM, Bes M, Meugnier H (2011). Species identification of staphylococci by amplification and sequencing of the tuf gene compared to the gap gene and by matrix-assisted laser desorption ionization time-of-flight mass spectrometry. Eur J Clin Microbiol Infect Dis.

[19] Loveday HP, Wilson JA, Pratt RJ, Golsorkhi M, Tingle A, Bak A (2014). epic3: national evidence-based guidelines for preventing healthcare-associated infections in NHS hospitals in England. J Hosp Infect.

[20] Chen YC, Chiang LC (2007). Effectiveness of hand-washing teaching programs for families of children in paediatric intensive care units. J Clin Nurs.

[21] Simonsen KA, Anderson-Berry AL, Delair SF, Davies HD (2014). Early-onset neonatal sepsis. Clinical microbiology reviews. Clin Microbiol Rev.

[22] Bhatta DR, Hosuru Subramanya S, Hamal D, Shrestha R, Gauchan E, Basnet S (2021). Bacterial contamination of neonatal intensive care units: How safe are the neonates?. Antimicrob Resist Infect Control.

[23] Akumatey S (2023). Ho Teaching Hospital NICU needs expansion to meet premature care demands. Accessed 7^th^ December.

[24] Afeke I, Moustafa A, Hirose M, Becker M, Busch H, Kuenstner A (2021). Draft Genome Sequences and Antimicrobial Profiles of Three Staphylococcus epidermidis Strains from Neonatal Blood Samples. Microbiol Resour Announc.

[25] National Library of Medicine-National Center for Biotechnology Information (NLM-NCBI) Basic Local Alignment Search Tool (BLAST).

[26] Ondov BD, Treangen TJ, Melsted P, Mallonee AB, Bergman NH, Koren S (2016). Mash: fast genome and metagenome distance estimation using MinHash. Genome Biol.

[27] Monsen T, Karlsson C, Wiström J (2005). Spread of clones of multidrug-resistant, coagulase-negative staphylococci within a university hospital. Infect Control Hosp Epidemiol.

[28] Liakopoulos V, Petinaki E, Efthimiadi G, Klapsa D, Giannopoulou M, Dovas S (2008). Clonal relatedness of methicillin-resistant coagulase-negative staphylococci in the haemodialysis unit of a single university centre in Greece. Nephrol Dial Transplant.

[29] Sabat A, Malachowa N, Miedzobrodzki J, Hryniewicz W (2006). Comparison of PCR-based methods for typing Staphylococcus aureus isolates. J Clin Microbiol.

[30] Davis MA, Hancock DD, Besser TE, Call DR (2003). Evaluation of pulsed-field gel electrophoresis as a tool for determining the degree of genetic relatedness between strains of Escherichia coli O157: H7. J Clin Microbiol.

[31] Johnson JK, Arduino SM, Stine OC, Johnson JA, Harris AD (2007). Multilocus sequence typing compared to pulsed-field gel electrophoresis for molecular typing of Pseudomonas aeruginosa. J Clin Microbiol.

[32] Trujillo S, Keys CE, Brown EW (2011). Evaluation of the taxonomic utility of six-enzyme pulsed-field gel electrophoresis in reconstructing Salmonella subspecies phylogeny. Infect Genet Evol.

[33] Blanc DS, Francioli P, Hauser PM (2002). Poor value of pulsed-field gel electrophoresis to investigate long-term scale epidemiology of methicillin-resistant Staphylococcus aureus. Infect Genet Evol.

[34] Soeorg H, Huik K, Parm U, Ilmoja ML, Metelskaja N, Metsvaht T (2013). Genetic relatedness of coagulase-negative Staphylococci from gastrointestinal tract and blood of preterm neonates with late-onset sepsis. Pediatr Infect Dis J.

[35] Kacica MA, Horgan MJ, Preston KE, Lepow M, Venezia RA (1994). Relatedness of coagulase-negative staphylococci causing bacteremia in low-birthweight infants. Infect Control Hosp Epidemiol.

[36] Foka A, Chini V, Petinaki E, Kolonitsiou F, Anastassiou ED, Dimitracopoulos G (2006). Clonality of slime-producing methicillin-resistant coagulase-negative staphylococci disseminated in the neonatal intensive care unit of a university hospital. Clin Microbiol Infect.

[37] Soeorg H, Metsvaht HK, Keränen EE, Eelmäe I, Merila M, Ilmoja ML (2019). Genetic Relatedness of Staphylococcus haemolyticus in Gut and Skin of Preterm Neonates and Breast Milk of Their Mothers. Pediatr Infect Dis J.

[38] Westberg R, Stegger M, Söderquist B (2022). Molecular Epidemiology of Neonatal-Associated Staphylococcus haemolyticus Reveals Endemic Outbreak. Microbiol Spectr.

[39] Widerström M, Monsen T, Karlsson C, Wiström J (2006). Molecular epidemiology of meticillin-resistant coagulase-negative staphylococci in a Swedish county hospital: evidence of intra-and interhospital clonal spread. J Hosp Infect.

[40] Takeuchi F, Watanabe S, Baba T, Yuzawa H, Ito T, Morimoto Y (2005). Whole-genome sequencing of staphylococcus haemolyticus uncovers the extreme plasticity of its genome and the evolution of human-colonizing staphylococcal species. J Bacteriol.

